# Attitudes towards telemedicine in ophthalmology: a population-based online survey in Germany

**DOI:** 10.1186/s12913-025-13491-1

**Published:** 2025-09-17

**Authors:** David J. Fink, Frank G. Holz, Robert P. Finger, Jan Henrik Terheyden

**Affiliations:** 1 Department of Ophthalmology, University Hospital Mannheim & Medical Faculty Mannheim, University of Heidelberg, Mannheim, Germany; 2 Department Of Ophthalmology Venusberg-Campus 1, University Hospital Bonn, 53127 Bonn, Germany

**Keywords:** Telemedicine, Tele-ophthalmology, Survey

## Abstract

**Background:**

Despite the increasing impact of telemedicine services on global healthcare provision, little data on acceptance and preference for telemedicine services at the population level are available to date. This is especially important for telemedicine in ophthalmology, where commercial telemedical applications are regularly offered outside of the state-funded health care system. Our study investigates the acceptability of tele-ophthalmology to the German population and associated sociodemographic factors.

**Methods:**

We conducted a cross-sectional survey via an online panel to assess attitudes towards telemedicine. In this survey, 1,008 individuals, demographically representative of the adult German population, were asked about their attitudes toward the acceptance of remote consultations and remote screening/monitoring in ophthalmology, as well as the motivators for telemedicine adoption. Associations with sociodemographic factors, including age, sex, household income and level of education, were investigated based on logistic regression models.

**Results:**

The mean age of the sample was 50 ± 16 years (range: 18–86 years). The acceptance of remote screening / monitoring (49.8%) was slightly higher than of remote consultations (45.7%). Younger age, higher income and reported visual difficulties were independent determinants favoring the acceptance of tele-ophthalmology. More than two in three participants (70.5%) reported easier or more flexible scheduling as motivators for the use of telemedicine, while fewer participants were motivated by cost savings (32.0%) or feedback reports (15.7%).

**Conclusions:**

Our survey suggests that one in two people in Germany has positive attitudes towards telemedicine in ophthalmology. Though next steps including a thorough technology acceptance assessment need to be performed, telemedicine services at the population level may need to specifically target older individuals, women and low-income populations to enable healthcare equity within telemedicine provision.

**Supplementary Information:**

The online version contains supplementary material available at 10.1186/s12913-025-13491-1.

## Introduction

The use of telemedicine services in high-income populations has dramatically increased over the past years, with a growing number of telemedicine services now offered in countries such as the United States and Germany [1, 2]. In low-and-middle income countries, many local or national tele-ophthalmology initiatives generate a significant impact on ophthalmic care delivery. For instance, much effort is put into establishing diabetic retinopathy screening programs via telemedicine applications in India, trying to lower access barriers, especially for rural populations [3]. In Columbia, telemedicine surveillance is successfully offered to improve care models for glaucoma patients [4]. However, telemedicine overall is still in its early stages [5]. Its implementation requires a framework enabling remote health assessments and treatment or surveillance decisions, which cannot be provided across medical specialties yet. Ophthalmology is a highly innovative medical specialty that encompasses all age ranges, and is regarded as a primary care specialty. Modern ophthalmology constitutes an ideal role model for the implementation of diagnostic procedures into telemedicine pathways since it routinely uses a broad spectrum of non-invasive imaging assessments and covers both acute and chronic care [6]. Besides this, ocular biomarkers derived from tele-ophthalmologic exams—subsumed under the term oculomics—may be useful in other medical fields, potentially enabling early detection and monitoring of systemic diseases, including neurodegenerative and cardiovascular conditions [7–10].

Despite the increasing impact of telemedicine services on global healthcare provision, little data on acceptance and preference for telemedicine services at the population level are available to date. Preliminary evidence suggests that the expansion of telemedicine can reduce no-show rates e.g. in patients with type 2 diabetes [11, 12]. However, the acceptability of tele-ophthalmology to the general population is largely unknown. The aim of our study was to systematically assess the acceptability of tele-ophthalmology to the German population and investigate associated sociodemographic factors to inform the design of telemedicine programs with respect to the populations they target.

## Methods

### Participants and survey design

Individuals from the general German population were recruited for a cross-sectional survey on health states and eye care provision in Germany via an online panel (SurveyEngine, Berlin, Germany). Individuals interested in survey research can register for the panel through an online platform and receive financial compensation via email for participating in given surveys. Participants were actively invited to match the age and sex distribution of the adult German population. Demographic data for this were obtained from the German national micro-census 2021 in adults and provided to the survey institute [13]. Survey invitations were distributed by the pre-provided quota and final replies demographically representative of the German population were provided to us, excluding duplicate responses based on internet protocol addresses and browser cookies. We have provided the CHERRIES checklist [14] as a supplementary file, summarizing the procedures used to obtain the results of our survey (Supplementary Table 3). All data were collected anonymously in September 2022 and the collection of items described in this manuscript followed no randomized order. The study was conducted in accordance with the Declaration of Helsinki and the institutional ethics committee at the University Hospital Bonn (Germany) approved the study (approval ID 255/22). Inclusion criteria were a participant age ≥ 18 years, sufficient German language proficiency, residence in Germany and internet access.

The survey included sociodemographic data (age, sex, marital status [categorical], level of education [categorical], monthly household income [categorical]), EQ-5D-5 L, participants’ self-reported general and ocular health (Likert-scale ratings), and previous use of the healthcare system (Table 1). Three multiple-choice items on the use of telemedicine were included, covering attitudes towards telemedicine and remote monitoring in the context of tele-ophthalmology:

**Table 1:** Responses to telemedicine items and sociodemographic characteristics of the sample (*n* = 1,008)

Telemedicine items				Mean ± SD or *n* (%)
Use of remote consultations				
	Yes			502 (49.8)
	No			483 (47.9)
Use of remote screening/ monitoring				
	Yes			461 (45.7)
	No			508 (50.4)
Motivators				
	Early availability of an appointment			508 (50.6)
	Time savings on the day of the appointment			469 (46.8)
	Scheduling flexibility (evening, weekends)			389 (38.8)
	Cost savings (travel expenses)			323 (15.4)
	Individual report			158 (7.5)
	Others			7 (0.7)
	None indicated			244 (24.3)
*Sociodemographic variables*				
Age, in years				50 ± 16
Sex	Female			500 (49.6)
	Male			501 (49.7)
	Diverse			3 (0.3)
	Missing			4 (0.4)
Education	Primary or lower secondary			41 (4.1)
	Upper secondary^*^			446 (44.2)
	Tertiary^#^			491 (48.7)
	Other^†^			14 (1.4)
	Missing			16 (1.6)
Employment	Employed or self-employed			567 (56.3)
	Retired			272 (27.0)
	Student			43 (4.3)
	Unemployed			49 (4.9)
	Other			73 (7.2)
	Missing			4 (0.4)
Living situation	Urban			638 (63.3)
	Rural			365 (36.2)
	Missing			5 (0.5)
Monthly household income	< EUR 1,300			128 (12.7)
	EUR 1,300 to < 1,700			114 (11.3)
	EUR 1,700 to < 2,600			242 (24.0)
	EUR 2,600 to < 3,600			155 (15.4)
	EUR 3,600 to < 5,000			234 (23.2)
	≥ EUR 5,000			94 (9.3)
	Missing			41 (4.1)
General health	Excellent			53 (5.3)
	Very good			230 (22.8)
	Good			437 (43.4)
	Fair			247 (24.5)
	Poor			41 (4.1)
Visual difficulties	No difficulties			648 (64.3)
	Some difficulties			327 (32.4)
	Large difficulties			27 (2.7)
	Cannot see			6 (0.6)

^*^ School education after age 16

^#^ University degree or similar

^†^ participants that reported school education after age 16 and an University or similar degree

1. Would you consider using remote medical consultations (e.g. phone or video consultations) / telemedicine in general? (referred to as “remote consultations” in the following sections) [Response options: “Yes”, “No”].
2. Would you consider using remote screening examinations / check-ups for eye disorders (e.g. devices in general practitioners’ offices or pharmacies)? (referred to as “remote screening / monitoring” in the following sections) [Response options: “Yes”, “No”].
3. Which factors might make remote medical consultations or telemedicine appealing to you? [Response options: “Getting an appointment quickly”, “Less time investment”, “Flexibility (evening, weekends)”, “Less costs (getting to the facilities)”, “Receiving a report”, “Education”, “Other”, “None”]

The items were drafted by two ophthalmologists with broad experience in public health research and underwent a debriefing process with selected individuals from the general public before they were launched within the survey. The questionnaire in the original German version and English translation can be found in the supplement (Supplementary Tables 1 and 2). A sample size of 1,000 participants from the general population was considered appropriate, reaching a margin of error (MOE) of 3% at the 95% confidence level according to the formula MOE = Z * (σ / √n).

### Statistical analysis

All data were analyzed descriptively followed by bivariable analysis and multivariable logistic regression modeling, to identify sociodemographic factors predicting acceptance and reasons for considering the use of telemedicine, with a specific focus on tele-ophthalmology. In the regression analysis we used partially binary independent variables, that concerning ‘higher’ monthly household income, the presence of visual difficulties and general health reduction needed to be derived from the summary of several self-reported participants’ characteristics (Tables 2 and 3). Statistical analyses were performed with SPSS Statistics, version 27 (IBM Corporation, Armonk, NY, USA), Winsteps, version 3.92.1 (Chicago, IL, USA) and R, version 4.2.2 (Vienna, Austria). P-values < 0.05 were considered statistically significant.

## Results

Out of 1,858 individuals who opened the survey, a total of 1,008 participants (54.2%) completed the survey, with a comparable distribution of male and female participants. Participants were on average 50 years old (range: 18–86 years; Table 1). One third reported at least some visual difficulties; only 4% reported a poor overall health status. A minor percentage of participants indicated having received diagnoses of cataract, glaucoma, age-related macular degeneration, or diabetic eye disease (6.9%, 4.3%, 4.9%, and 4.4%, respectively; 18.0 with any of these conditions).

The acceptance rates for asynchronous telemedicine were only slightly higher than for synchronous telemedicine consultations (49.8% and 45.7%, respectively). Our main outcome analysis suggests that younger age, higher household income and reported visual difficulties were associated with a higher willingness to use remote consultations, both in univariable and multivariable analyses (Tables 2 and 3). Level of education, health related quality of life or reported ocular diseases showed no association. Male participants were more likely to accept tele-ophthalmological screenings than female participants, however, there was no sex-related significant association concerning telemedicine in general. In a multivariate multiple logistic regression model, all factors that exhibited associations in the univariate model demonstrated correlations with increased receptivity to telemedicine applications, both overall and specifically in ophthalmology (Table 3).

**Fig. 1:**
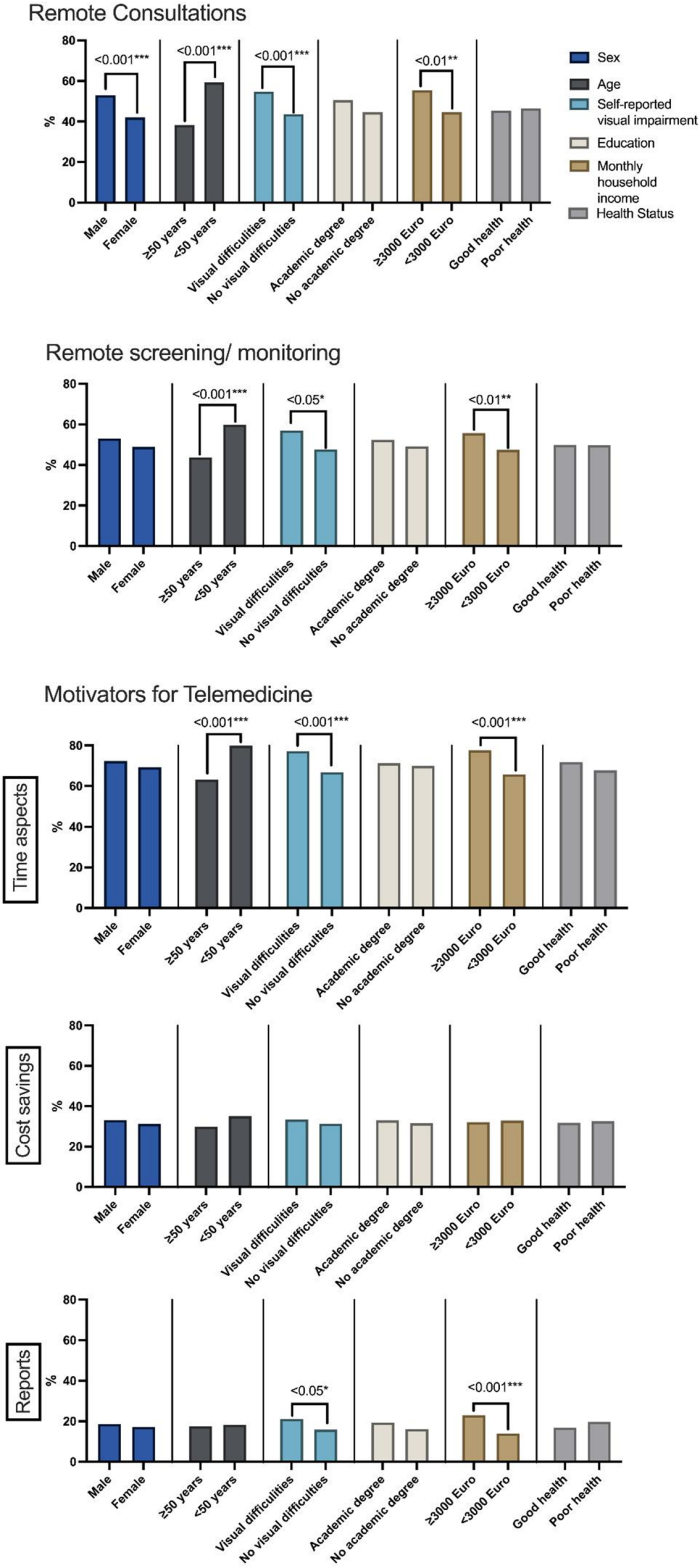
Sociodemographic factors associated with the main outcome items (*n* = 1,008). The figure shows the proportion of respondents with the respective characteristic who expressed an interest in teleconsultations or telemonitoring, or for whom the corresponding potential motivational factor is present. Only statistically significant results of a Chi^2^-test are depicted. For “Motivators for telemedicine”, a multiple response question, each graph shows the sociodemographic factors linked to one response category. The motivators ‘Early availability of an appointment ‘,‘Scheduling flexibility’ and ‘Time savings on the day of the appointment’ are compromised as category ‘Time’

The most frequently selected motivator to use telemedicine was the availability of appointments, as indicated by half of the participants (50.4%), followed by time savings (46.5%) and a possibly higher flexibility when scheduling appointments for example in the evening or during the weekend (38.6%) (Table 1). In total, 70.5% of the participants named one or more time-related motivators. The most frequent non-time associated reasons were possible cost savings (32.0%) and the provision of feedback reports (15.0%). For almost one quarter of the participants, no reason that could make them favor a tele-ophthalmological exam was indicated (24.2%). Higher income was associated with higher odds of reporting scheduling and the provision of individual reports (*p* < 0.01), younger age was associated with reporting scheduling and cost savings as motivators (*p* < 0.01; Fig. 1).

**Table 2:** Factors associated with higher overall openness to remote consultations and remote screening / monitoring in univariable binary-logistic regression models

Independent variable	Remote consultations		Remote screening / monitoring	
	OR [95% CI]	*p*-value	OR- [95% CI]	*p*-value
Age (per year)	**0.976 [0.968; 0.983]**	**< 0.001**	**0.970 [0.962; 0.978]**	**< 0.001**
Sex^1^ (Male)	1.177 [0.916;1.512]	0.202	**1.558 [1.209;2.009]**	**< 0.001**
Education^2^ (higher category)	1.139 [0.886; 1.465]	0.309	1.273 [0.987;1.640]	0.063
Marital status^3^ (married)	1.035 [0.805; 1.311]	0.789	1.149 [0.891:1.482]	0.285
Monthly household income^4^ (higher category)	**1.123 [1.033;1.221]**	**0.007**	**1.140 [1.047;1.241]**	**0.003**
Urban living area^5^	1.156 [0.892; 1.499]	0.272	0.924 [0.711; 1.202]	0.556
EQ-5D-5 L value	0.748 [0.418; 1.340]	0.329	0.668 [0.369;1.209]	0.182
General health reduction^6^	0.976 [0.740; 1.286]	0.863	0.976 [0.740; 1.286]	0.976
Visual difficulties^7^	**1.456 [1.120;1.893]**	**0.005**	**1.568 [1.203; 2.044]**	**< 0.001**
Consultation General Practitioner^8^	0.925 [0.718;1.193]	0.550	0.933 [0.722;1.206]	0.597
Consultation Ophthalmologist^8^	1.128 [0.873;1.458]	0.357	0.949 [0.733;1.228]	0.689

OR = Odds Ratio; OR related to ^1^ male vs female participants, ^2^ Tertiary education vs upper secondary/ primary and lower secondary education, ^3^ single/ divorced/ widowed vs married/ partnership, ^4^ Household income above the median of ≥ 2600 Euro vs income below 2600 Euro, ^5^ urban living area vs rural living area, ^6^ presence of a general health reduction “fair”/ “poor” vs “excellent”/”very good”/”good”, ^7^ presence of visual difficulties “some difficulties”/ “large difficulties“/”cannot see” vs “no difficulties”, ^8^ consultation during the last 12 months vs no consultation during the last 12 months; statistically significant associations are marked in bold

**Table 3:** Factors associated with higher overall openness to remote consultations and remote screening / monitoring in multivariable binary-logistic regression models

Independent variable	Remote consultations		Remote screening / monitoring	
	OR [95% CI]	*p*-value	OR- [95% CI]	*p*-value
Age (per year)	0.977 [0.969; 0.977]	**< 0.001**	0.971 [0.963; 0.979]	**< 0.001**
Sex^1^ (Male)	n.a.	n.a.	1.442 [1.101;1.887]	**0.008**
Monthly household income^2^ (higher category)	1.114 [1.022;1.215]	**0.014**	1.105 [1.011;1.208]	**0.028**
Visual difficulties^3^	1.438 [1.092;1.894]	**0.010**	1.469 [1.109; 1.947]	**0.007**

OR = Odds Ratio; OR related to ^1^ male vs. female participants, ^2^ Household income above the median of ≥ 2600 Euro vs. income below 2600 Euro, ^3^ presence of visual difficulties “some difficulties”/ “large difficulties“/ ”cannot see” vs. “no difficulties”

## Discussion 

Our survey confirms that discrepancies in attitudes towards telemedicine and tele-ophthalmology applications exist in the German population. 46% and 50% of our survey participants indicated to be in favor of asynchronous and synchronous telemedicine in the context of eye care, respectively. Age, household income and visual difficulties were significant predictors of attitudes towards tele-ophthalmology. Aspects related to time savings, flexibility and availability could increase participation rates in tele-ophthalmology services.

In line with previous data on telemedicine, younger survey participants were more open to tele-ophthalmology programs than older participants [15]. This finding may appear self-evident given the well-documented technology use gap among older individuals, but it carries important implications for the design of public health interventions targeting groups at risk of chronic conditions. Particularly, the availability of individualized caregivers may need to be specifically addressed by such campaigns. Besides limited familiarity with and availability of technology among older people, hearing and vision difficulties also present barriers in this target group and there is a need for low-barrier telemedicine services [16–18]. Despite decreasing technology familiarity with age, the interest in telemedicine is not necessarily age-dependent, which provides a strong rationale for actively including older populations into targeted telemedicine programs [18]. A strategy to increase the acceptance rates of telemedicine in older individuals could also include on-demand phone or in-person support for technology issues [18]. Apart from age, and in line with previous research, sex was also significantly associated with attitudes towards tele-ophthalmology in our sample [19, 20]. The underlying reasons for this association remain to be fully understood, as they are likely multifactorial and complex. One possible explanation may lie in differing self-perceptions between women and men: although no substantial sex differences in actual digital competence have been demonstrated, women tend to rate their own technological abilities lower than men [21]. Furthermore, it is conceivable that women, on average, may have a stronger preference for personal interaction in service settings, while men are more focused on functional and efficiency aspects, which could influence their acceptance of telemedical approaches [22].

Monthly household income was another significant predictor of positive attitudes towards tele-ophthalmology in our study, a relationship that has been the subject of debate in previous literature. While some data suggest higher telehealth adoption among lower-income groups, potentially due to greater access needs, other studies report a trend towards greater usage among higher-income individuals, possibly reflecting better access to technology and digital literacy skills required for telemedicine [23–25]. The cost structure of telemedicine services likely plays a pivotal role in shaping attitudes toward telemedicine among lower-income populations. Importantly, our survey participants were not primed with information regarding whether tele-ophthalmology services would be covered by health insurance, which may have influenced their responses. Nonetheless, income remains a major determinant of health inequalities in Germany. The significant association we observed between income and the acceptability of tele-ophthalmology services underscores the need for tailored telemedicine outreach strategies that specifically address barriers faced by lower-income groups (e.g. transportation costs not covered by insurances) [26]. Of note, our data did not show a significant association between the level of education and attitudes towards tele-ophthalmology, which is unlike previous reports [27, 28]. This may be related to ceiling effects of the education item used in our survey and requires further investigation.

Lastly, the presence of symptoms (“visual difficulties”) was associated with increased odds of positive attitudes towards tele-ophthalmology. Ongoing debates around long waiting times to obtain specialist appointments in Germany reflect this finding well if individuals assume that tele-ophthalmology appointments can be scheduled within shorter time intervals.

Of note, routine care does not yet incorporate the provision of tele-ophthalmology services in Germany. For example, only 10.7% of the German population have experiences with video consultations, which contrasts overall positive attitudes towards tele-ophthalmology in up to 50% of people who completed our survey [29]. Though in the context of the Covid-19 pandemic, prior studies of patient satisfaction in tele-ophthalmology from Anglo-Saxon countries demonstrate that telemedicine services can be successfully implemented, resulting in 78% to 88% of participating patients being open to further telemedical consultations afterwards [30, 31]. However, both studies did not evaluate patients’ perspectives on telemedicine prior to the teleconsultation. Future studies are needed to assess the impact of experiences with telemedicine influence acceptance rates.

The most widely considered argument in favor of telemedicine in our survey participants was the availability of appointments, which highlights current challenges regarding appointment waiting times in the German health care system. When deciding on a criteria catalogue for the introduction telemedicine application of to the statutory health insurance scheme in Germany, the impact on waiting times should not be neglected. While telemedicine can help reduce travel time and waiting time, the exact mechanisms are still unclear and may be related to the timing rather than the type of care provision.

Outside of countries that provide universal health care coverage, the most pressing causes for implementing telemedicine may be different from timing aspects. Data from the United States census suggest lower household income as one of the main predictors of telemedicine use. One quarter of the people with a household income below 25,000 USD attended a telehealth visit within the last 4 weeks, more than in any higher-income group investigated [32]. A possible explanation of this could be care paths implemented in low-income insurance schemes that include telemedicine services due to the lower associated costs. This example shows that the results from our survey can only be transferred to other settings even among high-income countries with precaution.

Strengths of our study include the relatively large sample size, which allows for a precise outcome estimate (margin of error of 3%); the availability of a wide range of sociodemographic factors, which we controlled for in the statistical models; and the population-based nature of our sample, which was demographically representative of the adult German population. However, our study also comes with several limitations. Firstly, sampling bias may have occurred since the sample was recruited and surveyed via an online platform, using a convenience sampling approach. The fundamental nature of internet-based surveys limits the generalizability to other populations as obviously individuals with access to technology and higher technology literacy are more inclined to participate in such survey formats. This may have affected the absolute attitudes but has less impact on the findings regarding sociodemographic associations of participants’ attitudes. We did not assess participants’ use of telemedicine services prior to our survey. Therefore, information bias may limit the interpretation of our research findings but could be in fact comparable to the application of telemedicine in public health settings. The aim of our study is exploratory in nature and intended to provide initial starting points for further quantitative and qualitative research on the real-world acceptance of tele-ophthalmology programs. Due to this, external validation of the items used to assess attitudes towards telemedicine is pending. Also, we did not test the participants’ knowledge of telemedicine and therefore cannot comment on their ability to correctly comprehend the items, which would have required qualitative work not possible with a sample recruited via an online platform. Lastly, our data collection was cross-sectional and it will be interesting to further investigate the development of attitudes with growing tele-ophthalmology experience in patients in the future.

In conclusion, our results demonstrate that nearly half of the population accept the concept of telemedicine service provision in eye care, for a variety of reasons. Based on our finding, any tele-ophthalmology services implemented in the German health system will have to be well-targeted and coordination specifically needs to address older individuals, women and low-income populations to prevent the emergence of a care gap compared with other groups.

## Supplementary Information

Below is the link to the electronic supplementary material.

Supplementary Material 1

## Data Availability

Data and material to reproduce the findings reported in this manuscript can be obtained from the authors upon reasonable request. They cannot be made publicly available because they have only agreed to collaborators of the research team to obtain the data in the informed consent document.

## Abbreviations

CI: Confidence interval
OR: Odds Ratio

## References

[1] Pylypchuck Y, Barker W. Use of Telemedicine among office-based physicians.; 2021. Accessed July 21, 2025. https://www.healthit.gov/sites/default/files/2023-04/DB65_TelemedicinePhysicians_508.pdf 39475515

[2] Heuer J, Osterwald A, Akmatov M et al. Telemedicine as an alternative access to outpatient health care by statutory health insurance physicians - trends in the period 2017 to 2021. 2023;23:1–21. 10.20364/VA-23.06

[3] Ramasamy K, Mishra C, Kannan NB, Namperumalsamy P, Sen S. Telemedicine in diabetic retinopathy screening in India. Indian J Ophthalmol. 2021;69(11). https://journals.lww.com/ijo/fulltext/2021/11000/telemedicine_in_diabetic_retinopathy_screening_in.12.aspx 10.4103/ijo.IJO_1442_21 PMC8725153 34708732

[4] Lozano AC, Serrano A, Salazar D, Rincón JV, Pardo Bayona M. Telemedicine for screening and follow-up of glaucoma: a descriptive study. Telemedicine e-Health. 2024;30(7):1901–8. 10.1089/tmj.2023.0676. 38662524 10.1089/tmj.2023.0676

[5] Garcia JP, Avila FR, Torres-Guzman RA, et al. A narrative review of telemedicine and its adoption across specialties. Mhealth. 2024;10:19–19. 10.21037/mhealth-23-28. 38689613 10.21037/mhealth-23-28 PMC11058596

[6] Keane PA, Sadda SR. Retinal imaging in the twenty-first century. Ophthalmology. 2014;121(12):2489–500. 10.1016/j.ophtha.2014.07.054. 25282252 10.1016/j.ophtha.2014.07.054

[7] Terheyden JH, Wintergerst MWM, Pizarro C, et al. Retinal and choroidal capillary perfusion are reduced in hypertensive crisis irrespective of retinopathy. Transl Vis Sci Technol. 2020;9(8):42. 10.1167/tvst.9.8.42. 32855888 10.1167/tvst.9.8.42 PMC7422770

[8] Langner SM, Terheyden JH, Geerling CF, et al. Structural retinal changes in cerebral small vessel disease. Sci Rep. 2022;12(1):9315. 10.1038/s41598-022-13312-z. 35662264 10.1038/s41598-022-13312-z PMC9166694

[9] Wagner SK, Fu DJ, Faes L, et al. Insights into systemic disease through retinal imaging-based oculomics. Transl Vis Sci Technol. 2020;9(2). 10.1167/tvst.9.2.6. 10.1167/tvst.9.2.6 PMC7343674 32704412

[10] Wagner SK, Hughes F, Cortina-Borja M, et al. AlzEye: longitudinal record-level linkage of ophthalmic imaging and hospital admissions of 353 157 patients in london, UK. BMJ Open. 2022;12(3):e058552. 10.1136/bmjopen-2021-058552. 35296488 10.1136/bmjopen-2021-058552 PMC8928293

[11] Khairat S, Yao Y, Coleman C, McDaniel P, Edson B, Shea CM. Changes in patient characteristics and practice outcomes of a tele-urgent care clinic pre- and post-COVID-19 telehealth policy expansions. Perspect Health Inform Managemen. 2022;19(Spring):1k. PMC9123528 35692856

[12] Sun CA, Perrin N, Maruthur N, Renda S, Levin S, Han HR. Predictors of follow-up appointment no-shows before and during COVID among adults with type 2 diabetes. Telemedicine e-Health. 2023;29(6):851–65. 10.1089/tmj.2022.0377. 36342782 10.1089/tmj.2022.0377 PMC10277979

[13] Federal Statistical Office of Germany (Destatis). Microcensus 2021. 2021. Accessed October 17, 2023. https://www-genesis.destatis.de/datenbank/online/statistic/12211/details

[14] Eysenbach G. Improving the quality of web surveys: the checklist for reporting results of internet E-surveys (CHERRIES). J Med Internet Res. 2004;6(3):e34. 10.2196/jmir.6.3.e34. 15471760 10.2196/jmir.6.3.e34 PMC1550605

[15] Scott Kruse C, Karem P, Shifflett K, Vegi L, Ravi K, Brooks M. Evaluating barriers to adopting telemedicine worldwide: a systematic review. J Telemed Telecare. 2018;24(1):4–12. 10.1177/1357633X16674087. 29320966 10.1177/1357633X16674087 PMC5768250

[16] Lam K, Lu AD, Shi Y, Covinsky KE. Assessing telemedicine unreadiness among older adults in the United States during the COVID-19 pandemic. JAMA Intern Med. 2020;180(10):1389. 10.1001/jamainternmed.2020.2671. 32744593 10.1001/jamainternmed.2020.2671 PMC7400189

[17] Anderson M, Perrin A, Smith A. Tech adoption climbs among older adults. 2017;17. https://www.pewresearch.org

[18] Mao A, Tam L, Xu A, et al. Barriers to telemedicine video visits for older adults in independent living facilities: mixed methods cross-sectional needs assessment. JMIR Aging. 2022;5(2). 10.2196/34326. 10.2196/34326 PMC9066341 35438648

[19] Reicher S, Sela T, Toren O. Using telemedicine during the COVID-19 pandemic: attitudes of adult health care consumers in Israel. Front Public Health. 2021;9. 10.3389/fpubh.2021.653553. 10.3389/fpubh.2021.653553 PMC8165259 34079784

[20] von der Groeben S, Czaplicki A, Hegerl U, Reich H. Telemedicine during the COVID-19 pandemic in Germany: results from three nationally representative surveys on use, attitudes and barriers among adults affected by depression. Internet Interv. 2023;32:100622. 10.1016/j.invent.2023.100622. 37091132 10.1016/j.invent.2023.100622 PMC10114311

[21] Hargittai E, Shafer S. Differences in actual and perceived online skills: the role of Gender ^*^. Soc Sci Q. 2006;87(2):432–48. 10.1111/j.1540-6237.2006.00389.x.

[22] Sánchez-Hernández RM, Martínez-Tur V, Peiró JM, Moliner C. Linking functional and relational service quality to customer satisfaction and loyalty: differences between men and women. Psychol Rep. 2010;106(2):598–610. 10.2466/pr0.106.2.598-610. 20524565 10.2466/pr0.106.2.598-610

[23] Karimi M, Lee EC, Couture SJ et al. National survey trends in telehealth use in 2021: Disparities in Utilization and Audio vs. Video Services. US Department of Health & Human Services, 2022.

[24] Harris A, Jain A, Dhanjani SA, et al. Disparities in telemedicine literacy and access in the United States. Plast Reconstr Surg. 2023;151(3):677–85. 10.1097/PRS.0000000000009939. 36730344 10.1097/PRS.0000000000009939

[25] Roberts ET, Mehrotra A. Assessment of disparities in digital access among medicare beneficiaries and implications for telemedicine. JAMA Intern Med. 2020;180(10):1386. 10.1001/jamainternmed.2020.2666. 32744601 10.1001/jamainternmed.2020.2666 PMC7400206

[26] Lampert T, Kroll LE, Kuntz B, Hoebel J. Health inequalities in Germany and in international comparison: trends and developments over time. J Health Monit. 2018;3(Suppl 1):1–24. 10.17886/RKI-GBE-2018-036. 35586261 10.17886/RKI-GBE-2018-036 PMC8864567

[27] Call VRA, Erickson LD, Dailey NK, et al. Attitudes toward telemedicine in urban, rural, and highly rural communities. Telemedicine e-Health. 2015;21(8):644–51. 10.1089/tmj.2014.0125. 25839334 10.1089/tmj.2014.0125

[28] Tipre M, Scarinci IC, Pandya VN, et al. Attitudes toward telemedicine among urban and rural residents. J Telemed Telecare. 2024;30(4):722–30. 10.1177/1357633X221094215 35578537 10.1177/1357633X221094215 PMC12947939

[29] Wolf A, Calmer B, Etgeton S et al. E-Health Compendium Trend Guide Digitale Gesundheit 2021. https://e-health-com.de/fileadmin/user_upload/dateien/TrendGuide/TrendGuide_2021_Digitale_Gesundheit.pdf Accessed 10 Aug 2024.

[30] Chen Y, Ismail R, Cheema MR, Ting DSJ, Masri I. Implementation of a new telephone triage system in ophthalmology emergency department during COVID-19 pandemic: clinical effectiveness, safety and patient satisfaction. Eye. 2022;36(5):1126–8. 10.1038/s41433-021-01528-8. 34035494 10.1038/s41433-021-01528-8 PMC8147902

[31] Kalra G, Williams AM, Commiskey PW, et al. Incorporating video visits into ophthalmology practice: a retrospective analysis and patient survey to assess initial experiences and patient acceptability at an academic eye center. Ophthalmol Ther. 2020;9(3):549–62. 10.1007/s40123-020-00269-3. 32535837 10.1007/s40123-020-00269-3 PMC7293175

[32] Lee EC, Grigorescu V, Enogieru I et al. Updated national survey trends in telehealth utilization and modality: 2021–2022 (Issue Brief No. HP-2023-09).; 2023. Accessed October 15, 2024. https://aspe.hhs.gov/sites/default/files/documents/3574fb7903ecce7ba0e1a61bf72ef7a1/household-pulse-survey-telehealth-covid-ib.pdf 38913813

