# Supplementary material for: Attitudes towards telemedicine in ophthalmology: a population-based online survey in Germany
Source: BMC Health Serv Res. 2025 Sep 17;25:1202. doi: 10.1186/s12913-025-13491-1 (PMC12445035; doi:10.1186/s12913-025-13491-1)
Supplement: Supplementary file 1 — Supplementary Material 1 [file 12913_2025_13491_MOESM1_ESM.docx]

**Attitudes towards telemedicine in ophthalmology: A population-based online survey in Germany**

David J. Fink^1,2^, MD; Frank G. Holz, MD^1^; Robert P. Finger^1,2^, MD, PhD; Jan Henrik Terheyden^1^, MD

^1^ University Hospital Bonn, Department of Ophthalmology, Bonn, Germany

^2^ Department of Ophthalmology, University Hospital Mannheim & Medical Faculty Mannheim, University of Heidelberg, Mannheim, Germany

**Supplement**

**Supplementary Table 1:** English translation of the questionnaire used in the study

|  |  |
| --- | --- |
| **Sociodemographic characteristics** | |
| What is your gender? | Male |
|  | Female |
|  | Other/Prefer not to say |
| How old are you? (in years) |  |
|  | Prefer not to say |
| What is your marital status? | Single |
|  | Married/Partner |
|  | Former relationship |
|  | Divorced |
|  | Widowed |
|  | Prefer not to say |
| Have you attended a school or other educational institution after the age of 16? | Education continued after age of 16 |
|  | Degree or equivalent professional qualification |
| What is your household’s monthly net income? | Up to 1300 euros |
|  | 1300 euros up to 1700 euros |
|  | 1700 euros up to 2600 euros |
|  | 2600 euros up to 3600 euros |
|  | 3600 euros up to 5000 euros |
|  | 5000 euros and above |
|  | Prefer not to say |
| What type of area do you live in? | Urban |
|  | Rural |
| **Health** | |
| How would you describe your overall health? | Excellent |
|  | Very good |
|  | Good |
|  | Fair |
|  | Poor |
| Do you have any difficulties with your vision, even when wearing glasses or contact lenses? | No difficulties |
|  | Some difficulties |
|  | Large difficulties |
|  | Cannot see |
| Do you have one or more of the following conditions? Please select all that apply | Cataract |
|  | Glaucoma |
|  | Age-related macular degeneration |
|  | Diabetic eye disease  No response |
|  |  |
| How often (in the last 12 months) did you see a GP? | Yes |
|  | Not at all |
|  | No response |
| How often (in the last 12 months) did you see an eye doctor? | Yes |
|  | Not at all |
|  | No response |
| **Telemedicine** | |
| Would you use telemedicine in general? | Yes |
|  | No |
|  | No response |
| What would be the reasons for you picking a telemedicine exam? | Getting an appointment quickly |
|  | Less time investment |
|  | Flexibility (evenings, weekends) |
|  | Less costs (getting to the facilities) |
|  | Receiving a report |
|  | Education |
|  | Other |
| Would you use telemedicine screenings for eye diseases (e.g. at GP, in pharmacies)? | Yes |
|  | No |
|  | No response |

*Notes:* All items were presented in a non-randomized order and were included in a survey on health states and eye care delivery in Germany. The survey was conducted in September 2022.

**Supplementary Table 2:** Survey items assessing attitudes towards telemedicine in German (original wording)

| **Item** | **Answer choices** |
| --- | --- |
|  |  |
| Würden Sie eine medizinische Fernbehandlung (z. B. über eine Telefon- oder Videosprechstunde, sogenannte Telemedizin) generell nutzen? | Ja |
|  | Nein |
|  | Keine Angabe |
| Würden Sie Vorsorgeuntersuchungen für Augenerkrankungen aus der Ferne in Anspruch nehmen (z.B. über Geräte in Hausarztpraxen oder Apotheken)? | Ja |
|  | Nein |
|  | Keine Angabe |
|  |  |
| Welche Gründe könnten für Sie die Nutzung einer medizinischen Fernbehandlung (Telemedizin) interessant machen? | Rasche Verfügbarkeit eines Termins |
|  | Zeitersparnis am Tag des Termins |
|  | Terminliche Flexibilität (z.B. Abendstunden, Wochenenden) |
|  | Kostenersparnis (z.B. bei der Anfahrt) |
|  | Individueller Ergebnisbericht |
|  | Erkrankungs-bezogene Schulungsangebote |
|  | Sonstige |
|  | Keine |
|  |  |

**Supplementary Table 3:** Checklist for Reporting Results of Internet E-Surveys (CHERRIES)^1^

| **Item Category** | **Checklist Item** | **Response** |
| --- | --- | --- |
| **Design** |  |  |
|  | Describe survey design | Sample demographically / sex-representative for the German population (targeted sample) |
| **IRB (Institutional Review Board) approval and informed consent process** | | |
|  | IRB approval Informed consent | yes, IRB approval was obtained (IRB University Hospital Bonn) |
|  | Informed consent | length of time: 30-60 min, data collection: anonymized, storage for up to 20 years, investigator: Robert Finger, purpose: understanding health states and experiences with eye care |
|  | Data protection | no collection of personal information |
| **Development and pre-testing** | |  |
|  | Development and testing | survey development based on qualitative work, extensive usability testing of functionality by staff members before survey launch |
| **Recruitment process and description of the sample having access to the questionnaire** | | |
|  | Open survey versus closed survey | closed |
|  |  |  |
|  | Contact mode | randomized invitation |
|  |  |  |
|  | Advertising the survey | direct e-mail to panel participants (panel participants from overall population [>10.000 individuals] --> random subsample invited as per quota) |
| **Survey administration** |  |  |
|  | Web/E-mail | online survey |
|  | Context | panel |
|  | Mandatory/voluntary | voluntary paid survey |
|  | Incentives | nominal incentives (<5 EUR) |
|  | Time/Date |  |
|  | Randomization of items or questionnaires | no randomization of items |
|  | Adaptive questioning | no |
|  | Number of Items | 5 |
|  | Number of screens (pages) | 30 |
|  | Completeness check | yes, age (18+), EQ5D completeness |
|  | Review step | none |
| **Response rates** |  |  |
|  | Unique site visitor | yes, cookies and panel level |
|  | View rate (Ratio of unique sur- vey visitors/unique site visitors) | not applicable, recruitment via panel |
|  | Participation rate (Ratio of unique visitors who agreed to participate/unique first survey page visitors) | 90.7% (1685/1858) |
|  | Completion rate (Ratio of users who finished the survey/users who agreed to participate) | 54.2% (1008/1858) |
| **Preventing multiple entries from the same individual** | |  |
|  | Cookies used | yes |
|  | IP check | yes |
|  | Log file analysis | no |
|  | Registration | yes, panel registration required. Only 1 participation per panel ID |
| **Analysis** |  |  |
|  | Handling of incomplete questionnaires | available datasets were all full datasets, |
|  | Questionnaires submitted with an atypical timestamp | not applied |
|  | Statistical correction | no weighting, invitation and selection of participants as per quota (German microcensus) |

^1^Eysenbach G. Improving the quality of Web surveys: the Checklist for Reporting Results of Internet E-Surveys (CHERRIES). J Med Internet Res. 2004 Sep 29;6(3):e34. doi: 10.2196/jmir.6.3.e34.
